# Association Between Substance Use and Trauma Outcomes in Adolescents

**DOI:** 10.5811/westjem.50673

**Published:** 2026-04-08

**Authors:** Stephen Sandelich, Angel Schuster, Ian Klansek, Olamide O. Olabamiji, Catherine Marco, Josh Glasser, Aleksandra E. Zgierska

**Affiliations:** * Penn State Milton S. Hershey Medical Center, Department of Emergency Medicine, Hershey, Pennsylvania; † Penn State Milton S. Hershey Medical Center, Department of Family and Community Medicine, Hershey, Pennsylvania

## Abstract

**Introduction:**

Adolescent substance use and substance use disorders are significant public health issues. Our goal was to evaluate the association between adolescent substance use, detected via blood alcohol levels and urine drug screens, and trauma-related outcomes at a Level I pediatric trauma center. Most of the literature is focused on adult trauma patients with limited data in the pediatrics.

**Methods:**

In this retrospective cohort study, we analyzed data from adolescent trauma patients 13–17 years of age presenting to a Level I pediatric trauma emergency department (ED). Demographic data, Injury Severity Score (ISS), intensive care unit (ICU) admission, hospital length of stay (LOS), and ED disposition were extracted from the Pennsylvania Trauma Systems Foundation Database Collection System, which includes comprehensive information on demographics, clinical characteristics, and outcomes of trauma patients. These data were compared between patients whose alcohol levels and urine drug screening were positive and negative. Our primary outcome measures were ISS and LOS in the hospital and ICU. Our secondary outcome measures were need for surgery, mortality, and disposition from the ED. Specific substances, including tetrahydrocannabinol (THC), benzodiazepines, and opioids, were further analyzed as drugs associated with these outcomes. We performed multivariate regression models to identify independent associations of blood alcohol levels or urine drug screen positivity with trauma severity and ICU admissions.

**Results:**

Among 405 adolescents who had toxicology testing done, 11/286 (3.8%) tested positive for alcohol, while 95/377 (25.2%) had positive urine drug screens predominantly for THC (19.9% of the 95 who had a positive screen). Blood alcohol level-positive patients demonstrated significantly lower ISS (P < .001), shorter ICU stays (P. < .01), and shorter overall hospital stays (P< .01) compared to blood alcohol level-negative patients. Conversely, benzodiazepine positivity was strongly associated with higher ISS, increased ICU admissions, and prolonged hospitalization stays. Multivariate analysis showed that older age was associated with increased ISS (β = 0.30 per year, P < .06) and ICU admission (OR 1.16, 95% CI, 1.04–1.28, P < .01). Blood alcohol level and most urine drug screen results were not independently associated with primary outcomes of ISS and LOS in the hospital and ICU, although benzodiazepine positivity was strongly associated with increased ISS (P < .001) and ICU admission (OR ≈ 30, P < .001).

**Conclusion:**

Adolescent trauma patients who were positive for benzodiazepines were associated with significantly worse outcomes, emphasizing the need for targeted screening and intervention strategies. Alcohol positivity was paradoxically associated with less severe trauma presentations. These findings highlight the complexity of substance use on adolescent trauma and underscore the importance of nuanced clinical assessments and targeted interventions addressing both substance use and underlying sociodemographic vulnerabilities.

## INTRODUCTION

Adolescent substance use and substance use disorders (SUD) remain a significant public health issue, contributing to considerable morbidity, mortality, and long-term health implications.^1^–^3^ In the United States, approximately 3.8 million adolescents 12–17 years of age reported past-year substance use, and 2.2 million met the criteria for a SUD.^4^ Although recent national data indicate a stabilization or decline in the rates of substance use among adolescents, mortality rates continue to rise.^5^,^6^ The early initiation of substance use has been linked to significant adverse outcomes immediately and later in life.^7^,^8^ Adolescents who begin using substances at younger ages face higher risks of developing a SUD, other mental health issues, and chronic health conditions.^9^ Individuals who started drinking alcohol before the age of 14 have higher levels of alcohol consumption and a greater likelihood of experiencing alcohol use disorder in adulthood compared to those who initiated alcohol use later.^10^

Adolescents who use substances are at a higher risk for trauma;^11^ substance use can also be a consequence of traumatic experiences, creating a cyclical relationship between the two.^12^ There is a positive relationship between substance use and worse trauma-related outcomes, including higher Injury Severity Scores (ISS), more extended hospital stays, and increased rates of intensive care unit (ICU) admissions.^13^,^14^ Many substances (eg, alcohol and opioids) impair cognitive and motor function, heightening the likelihood of accidents and serious injuries. Moreover, adolescents who use substances often require more intensive medical interventions when they present with trauma; this, in turn, places a significant burden on healthcare systems and increases overall treatment costs. Screening for substances should be done for all adolescents when presenting for trauma.^12^ Most of the literature is focused on the adult trauma population with limited data in the pediatric trauma population.

Emergency departments (ED) play a pivotal role as frontline points of care for adolescents presenting with acute injuries and trauma, especially those who do not engage with primary care. The ED is often the first and sometimes only contact adolescents have with the healthcare system, making it a suitable setting for the identification and management of substance use as the foundation for prevention and/or mitigating long-term harms related to SUD. While EDs can serve as crucial access points for immediate care and intervention, the specific outcomes related to substance use and trauma, as well as specific characteristics that may impact outcomes in adolescents, remain underexplored.

Previous studies have yielded conflicting findings regarding the impact of various substances on injury severity, hospital resource utilization, and survival outcomes. For instance, while alcohol intoxication is often presumed to worsen trauma severity, evidence from several trauma registry analyses indicates a paradoxical association: Intoxicated patients frequently present with less severe injuries, reduced ICU requirements, and lower immediate mortality compared to sober counterparts.^13^–^15^ In contrast, the impact of cannabis use remains ambiguous, with some large-scale studies reporting minimal effects on trauma severity and even associations with reduced mortality, while others suggest no protective benefit.^16^,^17^ Benzodiazepines, however, consistently emerge in literature as associated with worse clinical outcomes, including higher ISS, increased ICU admission rates, and prolonged hospital length of stay (LOS), likely due to their sedative properties and impact on cognitive and motor function.^18^,^19^

**Population Health Research Capsule**

What do we already know about this issue?

*For adolescents, there is an association between substance use and worse trauma-related outcomes*.

What was the research question?

*In adolescents, is use of specific substances associated with worse trauma-related outcomes?*

What was the major finding of the study? *Adolescents positive for benzodiazepines showed higher Injury Severity Scores (ISS) than those negative for benzodiazepines (mean ISS, 12.87 vs 10.01; P = .001)*.

How does this improve population health?

*This study highlights the association between adolescent substance use (particularly benzodiazepines) and increased trauma severity and healthcare use*.

Opioids and stimulants (such as methamphetamine and cocaine) demonstrate a varied clinical impact in trauma patients. Pre-injury opioid use often correlates with prolonged hospitalization and increased healthcare resource use but not necessarily increased mortality.^20^ Methamphetamine use similarly prolongs hospital and ICU LOS without consistently affecting mortality, whereas cocaine generally shows little measurable impact on trauma severity or survival outcomes.^21^ Given these contrasting and nuanced findings, it remains critically important to investigate substance-specific associations within adolescent trauma populations to inform clinical management and targeted intervention strategies more precisely.

In this study we aimed to fill critical knowledge gaps regarding the relationship between substance use and trauma-related outcomes in adolescents 13–17 years of age treated at a Level I pediatric trauma ED.

## METHODS

### Study Design and Setting

This study was a retrospective cohort analysis of data extracted from the Pennsylvania Trauma Systems Foundation Database Collection System (PTOS Trauma Registry), which includes comprehensive information on the demographics, clinical characteristics, and outcomes of trauma patients. The study population included adolescents 13–17 years of age who had presented to the ED at a Level I pediatric trauma center located in a suburban setting in the Mid-Atlantic region of the U.S. with traumatic injuries between January 1, 2018–August 31, 2023, for which either a urine drug screen or a blood alcohol level was obtained. They were identified through the PTOS Trauma Registry, which includes all trauma cases treated at the institution during the study period.

We excluded patients with missing or incomplete urine toxicology data. In addition, those who had received medications in the ED or prehospital that could have interfered with, or resulted in a positive result, on the ED-obtained urine drug screen were also excluded. Examples are patients who received benzodiazepine for anxiolysis or an opioid for pain management. Lastly, we excluded patients who were transferred to Penn State Medical Center after initial management at a different institution. The urine drug scene and blood alcohol level were obtained during initial trauma activation and evaluation.

The Institutional Review Board approved the study, and informed consent was waived due to the study’s retrospective nature. The study is reported according to Strengthening the Reporting of Observational Studies in Epidemiology STROBE guidelines.^22^

### Variables

The primary outcome variables included injury severity, measured by hospital admission and ISS, and LOS in the hospital and ICU. Secondary outcome measures included need for surgical intervention, mortality, and disposition from the ED (eg, admission to a medical or surgical ward, ICU, or discharge to home). Other secondary variables were collected as additional measures to obtain further information on factors that contributed to trauma outcomes; these variables collected included age, sex, race, and mechanism of injury (eg, motor vehicle collision, fall, gunshot wound).

### Statistical Methods

We used descriptive statistics to summarize the demographic, clinical, and outcome characteristics. Continuous variables were summarized as means (standard deviation [SD]), and categorical variables were presented as frequencies and percentages. Bivariate analyses (chi-square tests for categorical and independent-sample *t*-tests for continuous variables) compared outcomes between substance-positive and substance-negative patients. We used multivariate logistic regression to identify independent associators of severe trauma outcomes while adjusting for demographic variables, injury mechanisms, and other relevant confounders. All statistical analyses were performed using SAS statistical software v9.4 (SAS Institute Inc., Cary, NC). The statistical significance level was set at a *P* value of < .05.

### Criteria for Medical Record Review Studies

We reviewed the criteria for medical record review studies in emergency medicine published by Worster et al in 2005.^23^ Of the listed criteria, we met the following: inclusion and exclusion criteria defined; variables defined; data extraction form used to extract data from the PTOS Trauma Registry; medical record database identified; method of sampling described; management of missing data described; and, finally, the study was approved by our institutional review board.

## RESULTS

The final analytic cohort consisted of 405 adolescent trauma patients, predominantly male (66.4%), with a mean age of 15.4 years (SD 1.9). Most patients were White (74.1%), followed by Black (11.4%) and Asian (2.2%). Motor vehicle collisions were the leading mechanism of injury (57.0%), followed by falls (21.7%). Demographics of the study cohort are shown in Table 1.

The mean ISS for the cohort was 13.2 (SD 10.6) indicating moderate injury severity. The anatomical distribution of injuries varied, with external injuries being the most common (77.3%, n = 313). Head and neck injuries were frequently observed, with minor injuries (Abbreviated Injury Scale [AIS] score 2) accounting for 23.7% (n = 96) of this category. Thoracic and extremity injuries were also notably prevalent, with moderate severity scores (AIS 2 or 3) the most common.

Hospital admission was required for 78.8% (n = 319) of the patients. The ICU stays occurred in 27.7% (n = 112) of the cohort. Regarding disposition from the ED, 39.5% (n = 160) were admitted to medical/surgical units, 21.0% (n = 85) were admitted directly to the ICU, 18.3% (n = 74) went to step-down units, and 15.3% (n = 62) were transferred to operating rooms or preoperative holding. Only 5.4% (n = 22) were discharged directly home from the ED, with minimal cases transferred elsewhere (0.2%, n = 1) or resulting in mortality in the ED (0.2%, n = 1). Total ICU admissions (n = 112) are all admissions to the ICU whether the patient went straight from the ED to the ICU, or from the ED to the OR to ICU, and patients who were upgraded to the ICU at any time during their care. Emergency department disposition to the ICU (n = 85) only reflects patients who went directly from the ED to the ICU. The Figure presents ISS score and admission disposition for patients with a positive blood alcohol level or urine drug screen.

Blood alcohol level tests were conducted on 286 of the 405-patient sample. Of the 286 patients, 11 (3.8%) tested positive (Table 2). Urine drug screens were performed for 377 patients, of which 95 (25.2%) were positive for any substance. Of these, 95 (19.9%) tested positive for THC, followed by opioids (5.0%), benzodiazepines (3.2%), amphetamines (0.5%), cocaine (0.3%), and barbiturates (0.3%). There were just a few patients who had both a positive blood alcohol level and a positive urine drug screen. These patients did not have statistically significant outcomes compared to those with either a positive blood alcohol level or a positive urine drug screen; therefore, we did not report on this.

Patients positive for blood alcohol levels were older (mean age, 16.45 vs 15.53 years; < .10) compared to blood alcohol level-negative patients, although this difference was not statistically significant. However, blood alcohol level-positive adolescents had significantly lower ISS (8.55 vs. 13.88; *P* = .001), shorter ICU stays (mean ICU LOS, 0.55 vs. 1.72 days; *P* < .01), and shorter hospital stays (mean LOS, 2.55 vs. 6.06 days; *P* < .01) than blood alcohol level-negative patients. No significant differences were observed in ICU admission rates, social service consults, or ED disposition patterns in the blood alcohol level-positive patients.

Adolescents with positive urine drug screen were significantly older (mean age, 16.14 vs 15.05 years; *P* < .0001) and more likely to be non-White (*P* < .01). Urine drug screen-positive patients had similar ISS, hospital LOS, and ICU LOS compared to urine drug screen-negative patients (all *P* > .05). Additionally, no statistically significant differences were found in ICU admission rates, ED disposition, or social service consults for urine drug screen-positive patients.

Adolescents positive for benzodiazepines showed significantly higher ISSs (mean ISS, 12.87 vs 10.01; *P* = .001) and were significantly more likely to require ICU admission (75 vs 26.6%; OR 8.29, 95% CI, 2.18–31.25; *P* < 0.01) than those negative for benzodiazepines. Opioid-positive adolescents did not differ significantly from opioid-negative adolescents in injury severity, ICU LOS, hospital LOS, or ICU admission rates. However, a slightly higher proportion of ICU admissions was noted (36.8 vs 26.6%), although this difference did not reach statistical significance. Tetrahydrocannabinol-positive adolescents were significantly older (mean age, 16.33 vs 15.05 years; *P* < .001), more often male (76 vs 63.2%; *P* = .04), and non-White (*P* < .001). However, *THC* positivity did not significantly impact ISS (mean 11.99 vs 10.58; *P* = .32), hospital LOS, ICU LOS, ICU admissions, or social service consultations.

In multivariable regression, older age was independently associated with higher injury severity (β = 0.30 per year, *P* = .06) and increased likelihood of ICU admission (OR 1.16, 95% CI 1.04–1.28; *P* < .01). After adjusting for demographics, blood alcohol level positivity was not significantly associated with injury severity or ICU admission. Similarly, most urine drug screen categories were not associated with outcomes, with the exception of benzodiazepine positivity, which remained strongly associated with higher ISS (*P* < .001) and ICU admission (OR ≈ 30, *P* < .001).

## DISCUSSION

In this study we examined the association between adolescent substance use, detected via blood alcohol level and urine drug screen, and trauma outcomes in adolescents presenting to a Level I pediatric trauma center. The findings offer critical insights into the relationships between substance use and trauma severity, highlighting essential implications for clinical practice and public health policy. Our findings align with several prior studies that suggest a paradoxical effect of alcohol intoxication on trauma outcomes.

Contrary to our initial hypothesis, adolescents with positive blood alcohol levels demonstrated significantly lower ISS, shorter ICU LOS, and shorter hospitalizations compared to their non-impaired peers. Similar results have been observed in adult and adolescent populations, notably in traumatic brain injury cases, where alcohol intoxication correlated with reduced injury severity and lower mortality.^13^–^15^ This counterintuitive finding may reflect a combination of behavioral (less severe trauma mechanisms), biological (potential neuroprotective effects of alcohol), and clinical factors (heightened clinical suspicion and early aggressive management).^15^,^18^ These findings may appear counterintuitive, given alcohol’s known impairing effects; however, they do align with some previous data.^24^,^25^ Moreover, selective testing practices may introduce bias, with clinicians potentially opting to test less severely injured adolescents more frequently. Further research should explore standardized testing protocols and detailed injury context assessments to clarify alcohol’s influence on trauma outcomes.

In contrast, while overall positivity on urine drug screen was not consistently associated with significant differences in injury severity or hospital resource utilization, notable associations emerged when examining specific substances. Particularly, benzodiazepines were robustly associated with worse clinical outcomes, including significantly higher ISS, increased likelihood of ICU admission, longer ICU LOS, and distinct differences in ED disposition patterns. The sedative and cognitive-impairing effects of benzodiazepines likely contribute directly to higher trauma severity and complexity, exacerbating injury and complicating immediate clinical management.

Additionally, benzodiazepine-positive adolescents may represent a subgroup with pre-existing mental health conditions or polysubstance use behaviors that increase vulnerability and complicate discharge planning. Our results also support existing literature highlighting benzodiazepines as strongly associated with poorer clinical outcomes in trauma patients. Our findings are consistent with prior evidence linking benzodiazepine use to increased hospital resource requirements, greater injury severity, and higher ICU use, likely related to the cognitive and motor impairments induced by benzodiazepines.^18^,^19^ Despite this, we cannot extrapolate from our data that patients with a SUD with benzodiazepines are at higher risk of injury because urine drug screen is a screening tool, and quantitative testing is needed to confirm benzodiazepine use.

Opioid positivity was not independently associated with significantly worse trauma outcomes, although there was a non-significant trend toward increased ICU admissions among opioid-positive adolescents. This observation aligns with previous literature suggesting opioids may prolong hospitalizations without notably affecting mortality, reflecting potentially chronic use patterns and complex underlying health factors.^20^ Given the small sample size of opioid-positive patients, definitive conclusions are limited, and larger scale investigations are warranted.

Tetrahydrocannabinol positivity, despite being highly prevalent, showed limited associations with adverse clinical outcomes echoing recent large-scale studies indicating minimal clinical impact of cannabis use on trauma outcomes.^16^,^17^ While cannabis was the most commonly detected substance in our adolescent population, its presence did not correlate with increased injury severity or longer hospital stay, suggesting a relatively neutral effect compared to other substances like benzodiazepines or opioids. Tetrahydrocannabinol positivity should be interpreted with caution as a urine drug screen can remain positive for a significant amount of time after last use. Tetrahydrocannabinol-positive adolescents were significantly older and more frequently male and non-White, underscoring the need to understand better the broader sociodemographic factors influencing substance use patterns and clinical outcomes in adolescent populations.

Our multivariate analyses identified older age as being independently associated with trauma severity and ICU admission, highlighting broader sociodemographic disparities in adolescent trauma outcomes, consistent with literature identifying sociodemographic disparities as crucial determinants of trauma outcomes.^24^,^25^ Older adolescents likely engage more frequently in high-risk behaviors, leading to severe injuries. These findings emphasize the critical role of addressing broader social determinants of health, beyond substance use alone, when evaluating trauma risks and outcomes.

## LIMITATIONS

Several limitations of this study must be acknowledged. These data need to be interpreted with caution as a positive urine drug screen is not the same as acute intoxication. Depending on the substance, route, and chronicity of substance use, urine drug screens may be positive for a significant time after last substance use. Additionally, the retrospective design restricts causal inference, and reliance on clinical toxicology screening introduces potential misclassification bias due to timing variability, testing sensitivity, and clinical judgment in ordering tests.

We tried to minimize selection bias through inclusive cohort definitions. However, residual confounding likely persists due to unmeasured variables such as chronicity of substance use, precise timing relative to injury events, or underlying psychological conditions. We must also mention that different urine drug screen assays have different sensitivities for detecting benzodiazepines; therefore, it is possible for the urine drug screen to be falsely negative or positive. The urine drug screen at this Level I trauma center only screens for opioids, no opiates. Therefore, fentanyl and other synthetic opiates may not have been detected. Finally, the amphetamine assay of the urine drug screen is unreliable and many drugs, such as those used to treat attention deficit hyperactivity disorder, will yield a positive result.

Blood alcohol level and urine drug screen, per institution protocol, should be obtained for every trauma patient. However, actual practice of obtaining a blood alcohol level and urine drug screen for trauma cases may vary by clinician. There may have been bias at the clinician level, but there was no way to mitigate that bias in this study. In addition, we could not determine whether patients who had a positive urine drug screen in our dataset had prescriptions for these medications.

An additional limitation is the number of blood alcohol level-positive patients. Due to the retrospective nature of the study and that information was extracted from the Pennsylvania Trauma Systems Foundation Database Collection System, we could not extrapolate which criteria were used to determine when a patient would have a blood alcohol level (or urine drug screen) ordered. Only 286 of 405 patients had blood alcohol level testing sent. Reasons for not sending a blood alcohol level are unknown but may have been due to clinical judgement. Only 11 patients had a positive blood alcohol level making it difficult to draw significant conclusions on the relationship between positive blood alcohol level and injury severity. In this study, those with a positive blood alcohol level had lower injury severity as measured by ISS and hospital admission and LOS. However, these results may have been different if more patients with a positive blood alcohol level could have been included in the analysis. Levels of blood alcohol level-positivity were not correlated to severity of injury due to the small number of positives; having a larger sample of blood alcohol level-positive patients would have allowed for this correlation, which may have strengthened results.

Given the small sample size of 405 patients, our results should be interpreted with caution given the low number of positive results. Information was extracted from the Pennsylvania Trauma Systems Foundation Database Collection System, which provided data on all adolescents treated for trauma-related presentations during the study period. The Level I trauma center serves all Central Pennsylvania, which contains populations from rural, suburban, and urban settings. To strengthen the generalizability of our findings and clarify causal relationships, future research should prioritize prospective, multicenter studies with standardized toxicology screening protocols. Investigations into the effectiveness of ED-based interventions, such as brief motivational interviewing and structured referrals to substance abuse treatment, are essential. Addressing sociodemographic disparities and their influence on substance use and trauma outcomes should also be a focal point of future studies. Prospective, multicenter studies with standardized screening protocols are warranted to validate these associations.

The results of this study should be significant to those with a focus on policy and practice. Programs targeting broader social determinants, including mental healthcare access, community support, and socioeconomic interventions, could substantially reduce substance-related trauma risk among vulnerable adolescents. Policies and practices that focus on decreasing the use of alcohol and substances by adolescents are critical.

## CONCLUSION

Our study highlights critical associations between adolescent substance use, particularly benzodiazepines, and increased trauma severity and healthcare utilization. Systematic substance-use screening in pediatric trauma settings appears justified, particularly as a tool for identifying adolescents at higher risk for severe clinical outcomes and complex clinical trajectories. Our findings underscore the need for comprehensive clinical assessments and targeted interventions addressing both substance use and underlying sociodemographic vulnerabilities to improve outcomes among injured adolescents.

## Figures and Tables

**Figure 1:**
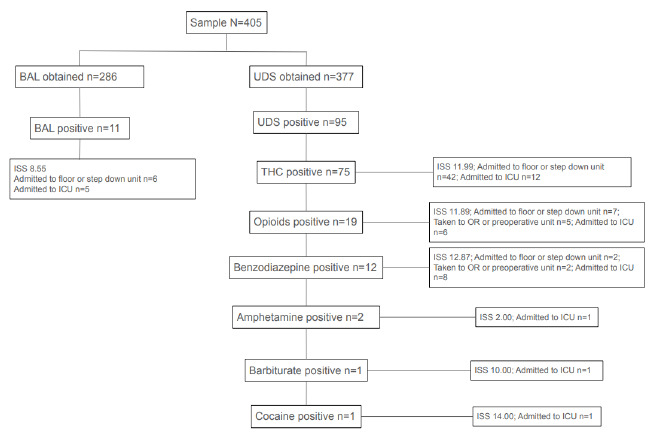
Injury Severity Score and admission disposition for patients with a positive blood alcohol level or urine drug screen in a study of the association between positive screening results and trauma severity, injury severity, and emergency department disposition. *BAL, blood alcohol level; ICU*, intensive care unit; *ISS*, Injury Severity Score; *OR*, operating room; *UDS*, urine drug screen.

**Table 1:** Baseline demographics of study cohort (N = 405) in a study of the association between blood alcohol level or urine drug screen, trauma severity, and Injury Severity Score.

	Mean	SD

Age	15.4	1.9

	Frequency	Percentage
Sex		
Female	136	33.6
Male	269	66.4
Race		
White	300	74.1
Black	46	11.4
Asian	9	2.2
Unknown	50	12.3
Primary Insurance		
Medicaid	103	25.4
Self-pay	52	12.8
Commercial managed care	97	23.9
Commercial indemnity	20	4.9
Other third party	133	32.8
Diagnosis		
Motor vehicle collision	231	57.0
Fall	88	21.7
Gunshot wound	32	7.9
Bike accident	18	4.4
Suicidal ideation	8	2.0
Fight	8	2.0
Animal bite	5	1.2
Fall off horse	4	1.0
Caught between 2 objects	4	1.0
Burn	1	0.2
Other/unknown	1	0.2
Injury Severity Score	13.2	10.6
Head Neck		
1	28	6.9
2	96	23.7
3	46	11.3
4	22	5.4
5	26	6.4
6	0	0.0
Face		
1	29	7.1
2	42	10.4
3	2	0.5
4	0	0.0
5	0	0.0
6	0	0.0
Thorax		
1	7	1.7
2	36	8.9
3	59	14.6
4	25	6.2
5	4	1.0
6	1	0.2
Abdomen		
1	4	1.0
2	50	12.3
3	32	7.9
4	19	4.7
5	6	1.5
6	0	0.0
Extremity		
1	3	0.7
2	99	24.4
3	71	17.5
4	0	0.0
5	0	0.0
6	0	0.0
External		
1	313	77.3
2	6	1.5
3	0	0.0
4	0	0.0
5	0	0.0
6	0	0.0
Need for Admission		
Yes	381	94.1
No	86	21.2
Need for ICU		
Yes	112	27.7
No	293	72.3
ED Disposition		
Med/surgery	160	39.5
ICU	85	21.0
Step down	74	18.3
OR/preop	62	15.3
Home	22	5.4
Transfer	1	0.2
Morgue	1	0.2

*ICU*, intensive care unit; *ISS*, Injury Severity Score; *OR*, operating room*; UDS*, urine drug screen; *SD*, standard deviation.

**Table 2:** Blood alcohol level and urine drug screen results in a study of adolescent trauma cases presenting to a Level I trauma center.

	Frequency	Percentage
Blood alcohol level (n = 286)		
Positive	11	3.8
Negative	275	96.2
Urine drug screen (n = 377)		
Positive	95	25.2
Negative	282	74.8
Tetrahydrocannabinol (n = 377)		
Positive	75	19.9
Negative	302	80.1
Cocaine (n = 377)		
Yes	1	0.3
No	376	99.7
Opioids (n = 377)		
Yes	19	5.0
No	358	95.0
Benzodiazepines (n = 377)		
Yes	12	3.2
No	365	96.8
Amphetamine (n = 377)		
Yes	2	0.5
No	375	99.5
Barbiturates (n = 377)		
Yes	1	0.3
No	376	99.7
